# Effects of probiotic supplementation with weight reducing intervention on anthropometric measures, body composition, eating behavior, and related hormone levels in patients with food addiction and weight regain after bariatric surgery: a study protocol for a randomized clinical trial

**DOI:** 10.1186/s40795-023-00717-w

**Published:** 2023-04-18

**Authors:** Fateme Ghafouri-Taleghani, Behnaz Abiri, Ali Zamanian, Atoosa Saidpour

**Affiliations:** 1 Department of Clinical Nutrition & Dietetics, Faculty of Nutrition and Food Technology, National Nutrition and Food Technology Research Institute, Shahid Beheshti University of Medical Sciences, Tehran, Iran; 2 Obesity Research Center, Research Institute for Endocrine Sciences, Shahid Beheshti University of Medical Sciences, Tehran, Iran

**Keywords:** Probiotic, Weight regain, Bariatric surgery, Eating behavior, Food addiction

## Abstract

**Background:**

One of the unfortunate events after bariatric surgery is the weight regain, which occurs in some patients. Food addiction is an eating disorder related to the brain-intestinal axis and can be effective in weight regain after bariatric surgery. In addition, the gut microbiome plays a vital role in eating behaviors, including food addiction. So, this study will aim to evaluate the effects of probiotic supplementation with a weight-reducing diet and cognitive behavioral therapy on anthropometric measures, body composition, eating behavior, and related hormone levels, leptin, oxytocin, and serotonin, in patients with food addiction and weight regain after bariatric surgery.

**Methods:**

We will carry out a triple-blinded randomized clinical trial for 12 weeks to evaluate the effect of probiotic supplementation with a weight-reducing diet and cognitive behavioral therapy on anthropometric measures, body composition, eating behavior, and related hormone levels including leptin, oxytocin, and serotonin, in patients with food addiction and weight regain after bariatric surgery.

**Discussion:**

Based on the available evidence, probiotic supplementation by modifying the intestinal microbiome can improve food addiction and subsequent weight loss.

**Trial Registration:**

Iranian Registry of Clinical Trials IRCT20220406054437N1 Registered on 2022–06-01.

**Supplementary Information:**

The online version contains supplementary material available at 10.1186/s40795-023-00717-w.

## Background

The growing prevalence of obesity in the world has made it a worrying epidemic [1]. Diet therapy, behavioral therapy, drug medication, and surgery treat obesity [2]. Currently, the most important, effective, safe, and long lasting treatment for severe obesity is bariatric surgery [3, 4]. However, not all patients undergoing bariatric surgery can maintain their weight loss, and some experience weight regains [5]. The prevalence of weight regain after bariatric surgery varies depending on the type of surgery, the length of time after surgery, and the definition of weight regain [5, 6]. Different factors affect weight maintenance or weight regain after bariatric surgery, including the type of surgery, eating behavioral disorders, body mass index, eating habits, anatomical changes of the gastrointestinal tract, hormonal changes, gut microbiome changes, access to healthy food, having exercise, and the existence of support groups [7–9]. With weight regain, diseases associated with obesity also return. It is also psychologically unbearable for the patient because the patient feels that he has lost his last way of treatment [6]. Eating disorders are the important factors affecting weight consequences after bariatric surgery, which need to be given more attention [10, 11]. One eating disorder that has recently come to the attention of researchers is food addiction. Food addiction is persistent, uncontrolled overeating of high-calorie foods (high in fat and sugar) associated with activating the brain s dopaminergic system or brain reward system [12]. Food addiction is also associated with other eating disorders such as binge eating and other mental disorders such as depression and anxiety [13–15]. The intestinal microbiome is one of the known influential regions that play an important role in the etiology of many diseases, including obesity; brain-intestinal axis signaling plays an important role in controlling food intake and energy homeostasis, followed by obesity [16]. Normal eating behaviors are coordinated by intestinal balance and extraintestinal hedonic and hemostatic mechanisms [17]. Growing studies on the role of intestinal microbiota in the etiology and progression of eating disorders have been done [18]. Intestinal bacteria can affect appetite control systems by producing metabolites such as lipopolysaccharide and flagellin in several ways: a) Effect on the secretion of digestive hormones such as cholecystokinin b) Effect on the central nervous system c) Increasing the permeability of the blood–brain barrier and increasing the effect of circulating cytokines on the appetite regulation center; Furthermore, intestinal bacteria affect the melanocortin system by producing peptides that mimic the function of neuropeptides such as α-MSH (α-melanocyte-stimulating hormone); bacteria also affect the appetite regulation system by producing neurotransmitters such as serotonin [18, 19]. There is also a correlation between mood, eating behavior, and intestinal microbiota, in that stress and depression increase the consumption of delicious foods; subsequently, it affects the growth of intestinal microbiota [20]. Also, autonomic changes, hormonal changes, and inflammation from stress and depression alter the gut microbiota [20]. Changes in the Brain-Gut-Microbiome system in food addiction shift the balance of the hedonic-homeostatic system toward a hedonic mechanism [17]. Hormones such as leptin, serotonin, and oxytocin, have an important role in the hedonic and homeostatic system and result from eating behaviors and food addiction [21–25]. Increased leptin concentrations are associated with addictive behaviors [26]. Also, intestinal microbiota, influenced by food intake, are effective in leptin expression and resistance [27–30]. Oxytocin is another hormone involved in food intake and eating behaviors [31]. Food intake activates oxytocin neurons in the hypothalamus, and fasting reduces oxytocin levels [32]. Oxytocin receptors are also present in many brain areas, including ventral tegmental and accumbens nuclei. They are involved in the brain reward system and hedonic pathway controlling appetite and food intake [32]. Oxytocin levels are closely related to intestinal microbiota (depletion of intestinal microorganisms leads to a significant reduction in oxytocin levels; also, Lactobacillus reuteri is involved in modulating oxytocin) [33–35]. Serotonin is another hormone that is effective in both the homeostatic and hedonic systems of food control [36]. Serotonin receptors are expressed in the dorsal striatum, an important area in eating behavior [36]. Activating the serotonin receptor reduces dopaminergic signaling and the desire for food intake [36]. The gastrointestinal tract contains a significant portion of serotonin, and the gut microbiota plays an important role in regulating serotonin; spore-forming bacteria lead to the synthesis of serotonin in enterochromaffin cells [37]. Serotonin is also involved in the development of food addiction; Low levels of serotonin in the brain lead to overeating delicious and sugar-rich foods, and increased blood glucose levels lead to increased absorption of the amino acid tryptophan and ultimately increase serotonin levels [38]. Bariatric surgery leads to anatomical, neurological, and physiological changes affecting the signaling of the homeostatic and hedonic systems, as well as changes in gastrointestinal hormones and intestinal microbiome, which profoundly affect eating behavior and subsequent weighting consequences [39]. Few interventions have been performed to deal with weight regain, mainly in medication, reoperation, and behavioral therapy. Researchers are also looking for ways to deal with regaining weight after bariatric surgery. Various studies have been performed on the effectiveness of probiotics on anthropometric indices, hormone levels, and eating behavior In obese people without surgery [40–43]. To date, no study has been performed on the effect of probiotics on people with food addiction and weight regain after bariatric surgery. The prevalence of food addiction in people who have undergone bariatric surgery is reported to be between 14–58% [44, 45]. Due to the significant prevalence of food addiction in patients undergoing bariatric surgery and its effect on the weight consequences of these patients, as well as changes in the intestinal microbiome and the role of the intestinal microbiome in hormonal changes and its relationship with food addiction, and, there are several studies on the effectiveness of probiotics in intestinal microbiota balance and improving food addiction and weight management in obese patients without bariatric surgery, therefore In this clinical trial study, the effect of probiotic supplementation on the improvement of outcome measures of weight control and levels of leptin, oxytocin, serotonin, followed by the improvement of food addiction and weight management of patients with food addiction and weight regain after bariatric surgery has been surveyed.

### Objectives and hypothesis

This study aims to investigate the effect of probiotic supplementation with a weight-reducing diet and psychotherapy intervention on anthropometric measures, body composition, eating behavior, and related hormone levels (leptin, serotonin, oxytocin) in patients with food addiction and weight regain after bariatric surgery.

## Methods and design

### Study design

**Fig. 1:**
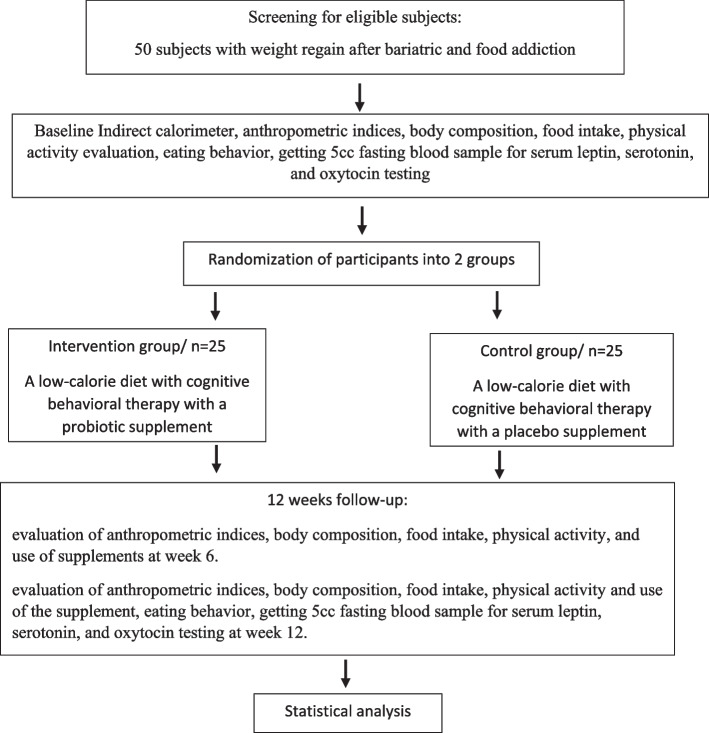
Protocol flow diagram; we will carry out a 12-week randomized controlled trial to determine the effects of probiotic supplementation with weight reducing diet on anthropometric measures, body composition, eating behavior, and related hormone levels in patients with food addiction and weight regain after bariatric surgery

**Fig. 2:**
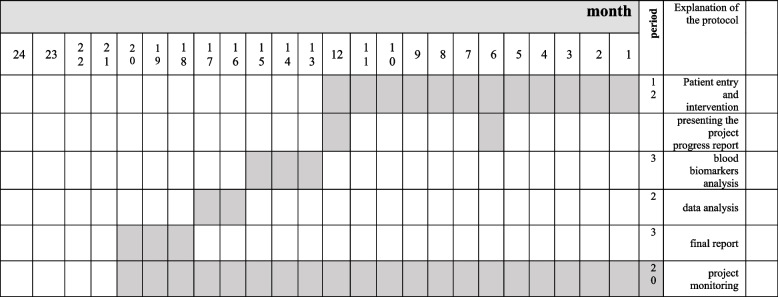
Timeline of the study; we expect the duration of the trial will be 20 months

We will conduct a 12-week triple-blind randomized clinical trial. The flow chart of the study is presented in Figs. 1 and 2. This intervention will be performed in the nutrition clinic of Shahid Beheshti University of Medical Sciences in Tehran, Iran, to evaluate the effect of probiotic supplementation with a weight-reducing diet and psychotherapy on anthropometric measures, body composition, eating behavior, and related hormone levels in patients with food addiction and weight regain after bariatric surgery. The protocol is written in line with the Standard Protocol Items Recommendations for Interventional Trials (SPIRIT) checklist (Additional file 1) [46].

### Sample size

The number of samples required for this study was calculated based on weight as a main dependent variable. The results of a previous study were used to calculate the sample size [47]. Sample size was calculated with α = 0.05 and power of 80% (1-β = 0.8). the sample size in each group was determined to be 20 patients; assuming a drop of 20%, 25 patients will be admitted to each group.

$$\begin{array}{c}\mathrm S1=2.8\\\mathrm S2\;=2.9\\\mathrm{Sp}\;=\;8.125\\\mathrm\mu1-\mathrm\mu2=2.5\\\end{array}$$

$$\begin{array}{c}\mathrm n=\frac{2\mathrm{Sp}^2\left({\mathrm Z}_{1-{\displaystyle\frac{\mathrm a}2}}+{\mathrm Z}_{1-\mathrm\beta}\;\right)^2}{\left(\mathrm\mu1-\mathrm\mu2\right)^2}\end{array}$$

### Study population

Patients with weight regain after bariatric surgery, and food addiction will be recruited from the Obesity Surgery Center of Sina Hospital and Rasoul Akram Hospital in Tehran.

Weight regain in this study was defined as: At least 18 months have passed since the surgery, and at least 10% of the lowest weight has returned. The diagnosis of food addiction will be based on the Food Addiction Questionnaire (Modified Yale Food Addiction Scale; mYFAS 2.0) [48]. Subjects who meet the inclusion criteria will be thoroughly informed about the protocol of the study. In addition, each participant will sign the informed consent form.

### Inclusion and exclusion criteria

For this study, 50 patients with weight regain and food addiction and with the following inclusion criteria will be included in the study: aged 18–50 years; BMI ≥ 25 kg/m2; willingness to participate in the study; not having diseases such as cancer, thyroid, renal or liver (except fatty liver); non-pregnant or lactating or menopause or professional athlete; not use of any antibiotics in the last three weeks and any protein or probiotic supplement in the last month.

Patients will be excluded if: taking any antibiotics during the study; using probiotic products (probiotic supplements, yogurt, cheese, cakes, biscuits, and probiotic pasta) or protein supplements during the study; using weight-loss or appetite-suppressing medications; pregnancy; consuming less than 90% of the supplements prescribed during the study Also, we will exclude participants who refuse to continue the study.

### Randomization

This study is a triple-blind randomized controlled clinical trial. A simple method has been used for randomization. The randomization unit is Individual. Excel software has been used for randomization. Using unique codes as ID numbers, using random numbers generated by the software, participants were randomly assigned to the same volume in the intervention and placebo groups. (Description of randomization by Excel software: In the first step, we generated 50 random numbers according to the sample size, in the next step, we obtained the rank of these numbers obtained in the first step, and in the third step, we divided these numbers by 25, in the fourth step, the numbers that fell between zero and one were in the intervention group and the rest in the placebo group) The primary service method was used for allocation concealment, and someone outside our study performed allocation concealment and labeling of supplements.

### Implementation

Potential participants will be invited to the study by FGT and AZ. The sequencing will be generated by AS, who is not going to assign participants into the study.

### Intervention

For all participants in this study, the standard hypocaloric diet (-300 to -500 kcal from total energy requirement) is calculated based on basal metabolism (use of indirect calorimeter) and level of physical activity. The diet in each group will consist of 45% carbohydrates, 25% protein, and 30% fat. For all participants in the study, ten one-hour online individual sessions, once a week, will be performed cognitive behavioral therapy (CBT) intervention by a psychologist. This study will randomly divide participants into groups receiving a probiotic supplement or placebo. All participants are asked to take 2 capsules daily (after breakfast and after lunch) for 12 weeks. Capsules are given to participants at the beginning of the study and in the sixth week of the study, and they are asked to return the unused capsules. To follow the participants and ensure their adherence to diet and receive supplements, participants will be contacted by telephone once a week. Also, the participants will be followed up and evaluated in person at the end of the sixth and twelfth weeks. Because multi-strain probiotics are more effective than single-strain probiotics [49, 50], multi-strain probiotics will be used in this study. Each probiotic capsule contains Lactobacillus acidophilus (1.8 × 109 CFU / capsule), Bifidobacterium bifidum (1.8 × 109 CFU / capsule), Bifidobacterium lactis (1.8 × 109 CFU / capsule), Bifidobacterium longul / C, 1.8 (1 × 109 CFU / capsule), Lactobacillus reuteri (1 × 109 CFU / capsule), magnesium stearate and maltodextrin. The placebo capsule contains 300 mg of starch. The capsules are similar in shape, size, and color and are provided by Takgen Zist Company in Iran.

### Adherence

To follow the participants and ensure their adherence to diet and receive supplements, participants will be contacted by telephone once a week. At every phone call, we will make sure if the cases are still in the study and they do not have exclusion criteria. Also, a 24-h recall is taken from patients on each phone call to ensure adherence to the diet. At the end of the sixth and twelfth weeks, the participants are followed up and evaluated in person. Individuals are asked to return unused supplements; if less than 90% of supplements are taken, the person will be excluded from the study. Data from patients who are more than 90% consistent with the intervention will be analyzed.

### Study outcomes

The primary outcomes of this clinical trial are the changes in anthropometric indices (including weight, hip circumference, waist-to-hip ratio, fat mass, muscle mass) and food addiction score. Secondary outcomes are changes in eating behavior and serum levels of leptin, serotonin, and oxytocin.

If the patient has inclusion criteria, a consent form and personal information sheet will be completed for them.

Food addiction will be diagnosed using a Modified Yale Food Addiction Scale Version 2.0 questionnaire. In this questionnaire, 12 criteria are examined. Both the symptom count score and the clinical significance criterion are used. For the "diagnosis" scoring option, a participant can meet for mild, moderate, or severe food addiction. At the beginning of the study, in the middle of the study (sixth week), and at the end of the study (twelfth week), anthropometric measurements, body composition, and calculation of body mass index will be done for each person. A digital scale will measure weight with an accuracy of 0.1 kg, with minimal clothing and no shoes. Height, waist, and hip circumference will be measured with an inelastic meter with an accuracy of 0.5 cm. Waist circumference will be measured at a distance between the lowest rib and iliac bone and hip circumference at the highest hip protrusion. Body mass index and waist-to-hip ratio are obtained by calculation. Body fat mass and muscle mass will be measured by bioelectrical impedance analysis (BIA). The participants' physical activity level is assessed using the standard questionnaire of activities (metabolic equivalents task) (MET) at the start, the sixth week, and the end of the study. Eating behavior is assessed using the Three-Factor Eating Questionnaire (TFEQ-R18) at the beginning, sixth week, and end of the study. Three 24-h food recall (2 regular days of the week and one day off) will be taken from participants at the beginning, the sixth week, and end of the study, also, a 24-h recall once a week, with telephone calls with patients. To measure blood biochemical parameters, at the beginning and end of the study, after 12–14 h of night fasting, five cc of venous blood samples will be taken from the participants. Blood samples taken in tubes containing sodium citrate anticoagulants will be collected and centrifuged in the laboratory of the Shahid Beheshti Nutrition Faculty. Serums will be stored at -80 °C until the tests are performed. Serum levels of leptin, serotonin (LDN ELISA Kit, Germany), and oxytocin (Crystal Day ELISA Kit, Shanghai) will be measured by the ELISA method with the human kit with the intra-assay and inter-assay CV of < 10.8% (serotonin), < 12% (leptin), and < 6.8% (oxytocin) kits in the laboratory of the Nutrition Institute of Shahid Beheshti University of Medical Sciences.

### Statistical analysis

In this study, data analysis will be performed by using SPSS version 21.0 (SPSS Inc, Chicago, Illinois) software. A paired t-test will be used to compare the mean of quantitative variables with normal distribution in each group between the beginning and the end of the study. The t-test will be used to compare the mean between the two groups at the beginning and end of the study. In the case of quantitative variables with non-normal distribution, Wilcoxon and Mann–Whitney tests are used, respectively. A repeated ANOVA test will be used for the variables measured three times during the study. Covariance analysis will be used to eliminate the effect of quantitative confounding factors. Quantitative confounding factors are physical activity and baseline values of the biochemical markers. A Chi-square test will be used to compare the qualitative variables between the two groups, and regression analysis will be used to eliminate the effect of qualitative confounding.

### Ethical considerations

Patients with weight regain after bariatric surgery, and food addiction who meet the inclusion criteria will be thoroughly informed about the study's protocol. The protocol of this study was approved by the ethics committee of Shahid Beheshti University of Medical Sciences and conformed with the declaration of Helsinki (approved number IR.SBMU.NNFTRI.REC.1400.099).

### Modification of study

Any modifications to the protocol, such as changes in the study objectives, study design, study method, study intervention, or sample size that affect the interests or safety of the patient, will be agreed upon by NNFTRI (National Nutrition and Food Technology Institute) and approved by the ethics committee of Shahid Beheshti University of Medical Sciences before implementation. NNFTRI will agree upon minor corrections that do not affect the way of the study.

## Discussion

Weight regain after bariatric surgery is one of the unpleasant events. The prevalence of weight regain depends on the type of surgery and how long it has been since surgery [5, 6]. Eating behaviors are one of the reasons for weight regain after bariatric surgery [7–11]. Food addiction is one of the eating behaviors that are directly related to the brain-intestine- axis. Recently, growing studies on the role of intestinal microbiota in the etiology and progression of eating disorders are underway [18]. There is a correlation between mood, hormonal changes, stress-induced inflammation, autonomic changes, eating behavior, and intestinal microbiota. It has been suggested that some bacterial species may cause dysregulated eating [20]. Intestinal microbiota affects eating behaviors by affecting the homeostatic and hedonic systems. However, in food addiction, a change in the Brain-Gut-Microbiome (BGM) system shifts this balance to hedonic mechanisms [17]. Hormones such as leptin, serotonin, and oxytocin, have an essential role in the hedonic and homeostatic system and result from eating behaviors and food addiction [21–25]. Bariatric surgery leads to anatomical, neurological, and physiological changes affecting the signaling of the homeostatic and hedonic systems, as well as changes in gastrointestinal hormones and intestinal microbiome, which profoundly affect eating behavior and weighing consequences [39].

It seems based on the available evidence, Probiotic supplementation by modifying the intestinal microbiome can lead to the improvement of food addiction and subsequent weight loss. Therefore, this study aimed to evaluate the effect of probiotic supplementation on the improvement of food addiction and its related hormones and the improvement of anthropometric indices in patients with food addiction and weight regain after bariatric surgery.

### Trial status

This trail is in the enrolment stage.

### Protocol version

Recruitment began on June 2022 and is expected to be completed on June 2023.

## Supplementary Information

**Additional file 1.** SPIRIT Checklist for *Trials.*

## Data Availability

Not applicable.

## Abbreviations

mYFAS: Modified yale food addiction scale
MET: Metabolic equivalent of task
TFEQ-R18: Three-Factor Eating Questionnaire
